# Providing eye care education for pharmacists

**Published:** 2023-05-22

**Authors:** Boateng Wiafe

**Affiliations:** Technical Advisor: Operation Eyesight Universal, Accra, Ghana.

**Community pharmacies are often the first place people will go to for help with an eye condition, so it makes sense to enhance their knowledge of eye care.**

Pharmacists play an important role in eye care. Many patients will go to their community pharmacy to seek over-the-counter or self-care medication for eye conditions. Some prescribed systemic medications may also contribute to eye problems (see article on page 14), so there really is a need to have a very close relationship between eye care practitioners and pharmacists.

Community pharmacies are often the first place many patients with eye problems will go to for help, especially in low- and/or middle-income countries, such as Ghana. So it is useful to empower the people who work there with basic knowledge of common eye disorders.

In 2018, I was invited to present at a series of workshops for pharmacists, hosted by Novartis Ghana. We spent time discussing what might bring the patients to pharmacists. These were the most common reasons shared by the pharmacists:

1. Red painful eye
2. Eye injuries
3. Itchy eyes
4. Not seeing well, both near and distance
5. Glaucoma
6. Growths and swellings
7. Various childhood conditions.

I discussed the following:

1. The normal and abnormal eye
2. The signs and symptoms of common eye conditions, the rationale for their management
3. The practice limitations of pharmacists.

Pharmacists’ role is to provide first aid and then refer, especially for eye injuries. As a rule of thumb, **any patient presenting with pain in the eye should be referred for further investigation immediately**.

## Health promotion

Pharmacists also play an important role in health promotion by providing patient education and counselling. Although pharmacists are not experts in treating eye conditions, they can give useful advice based on patients’ presenting symptoms and whether they came to purchase prescription or over-the-counter medicines. Pharmacists can also explain how the medicines should be used. 

For example:

- Counsel people with diabetes about the importance of having regular eye checks, so that any early signs of diabetic retinopathy can be detected.
- When refilling eye drop prescriptions for patients with glaucoma, ask them to encourage other family members to get their eyes checked
- Encourage parents to take their children for a sight test once a year

General eye messages to help people avoid vision loss and maintain good eyesight can also be delivered by pharmacists – whether in person or via posters and or leaflets. For example^1^:

- Your eyes are an important part of your health
- Have a comprehensive eye examination at least every two years
- Keep your blood sugar levels within a healthy range
- Know your family eye health history
- Eat right to protect your eyes
- Maintain a healthy weight
- Wear protective eye wear and do not leave children to play unsupervised by an adult
- Quit smoking or never start smoking.

**Top tips when treating common eye conditions with over-the-counter medications**

- **Avoid prescribing steroid drops if someone has a red, painful eye.** Steroids are prescribed *only* by the ophthalmologist. For example, if the person has a corneal ulcer, steroids can weaken the immune response, which will worsen the infection. (What other reasons might there be?) Topical steroid drops can cause an increase of intraocular pressure and can lead to secondary, steroid-induced glaucoma. Advise the patient to visit the nearest clinic for further management.
- **In case of an injury, never instil or prescribe any eye medication in the affected eye.** Refer immediately. This is an emergency.
